# Supplementary material for: Increased ectodysplasin-A2-receptor EDA2R is a ubiquitous hallmark of aging and mediates parainflammatory responses
Source: Nat Commun. 2025 Feb 23;16:1898. doi: 10.1038/s41467-025-56918-3 (PMC11847917; doi:10.1038/s41467-025-56918-3)
Supplement: Supplementary file 1 — Supplementary Information [file 41467_2025_56918_MOESM1_ESM.pdf]

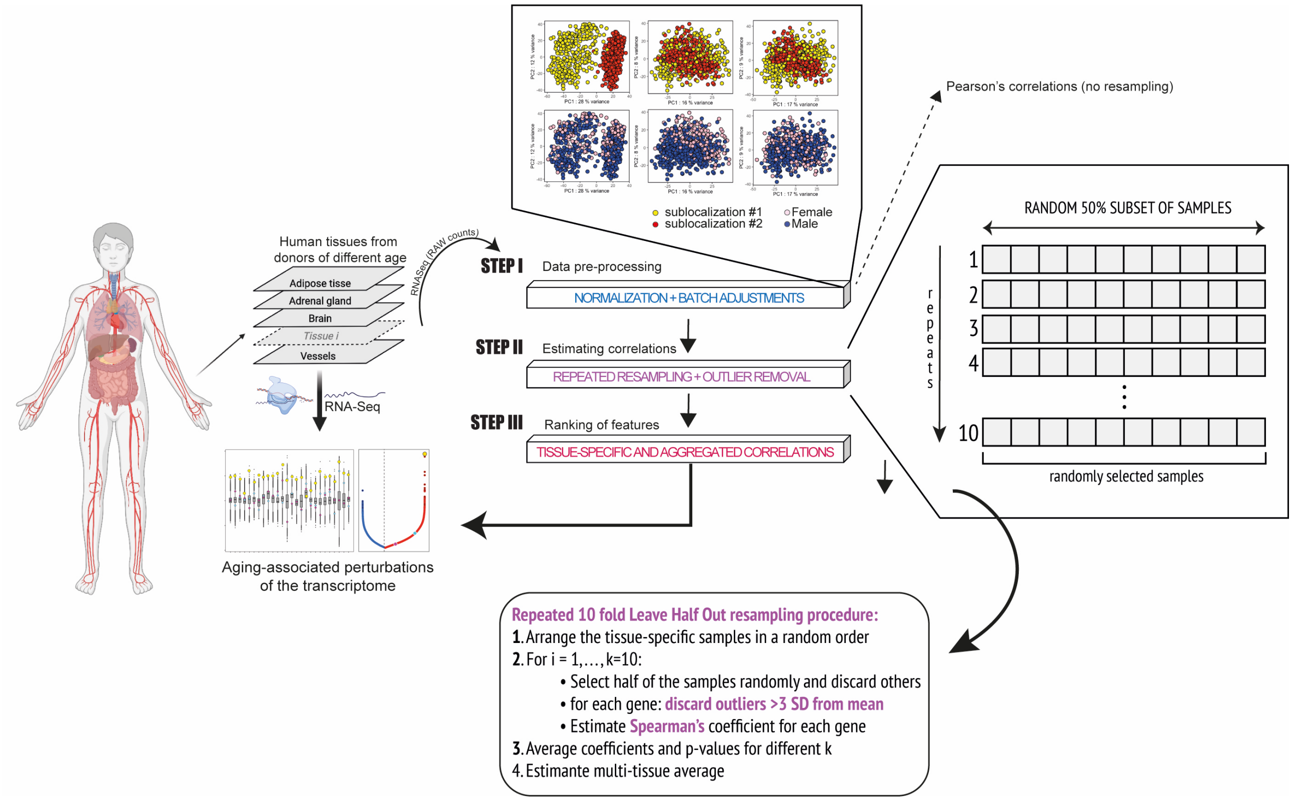

Multi-step workflow for correlation analysis between gene expression and age

Illustrated is the multi-step process used to ensure robust correlation results between gene expression and age across tissues. The workflow includes: 1) Data Pre-processing and batch effect removal 2) Leave-Half-Out (LHO) resampling of the data into 10 subsets per tissue, each containing 50% of the samples, to reduce the impact of outliers. Next, remaining outliers are discarded, where samples exhibiting expression values deviating more than three standard deviations from the mean for each given gene are excluded; 3) Spearman's rank correlations are computed for each gene across the subsets, and the results are averaged for each tissue. Subsequently, mean correlations across tissues are calculated to provide a comprehensive result. This approach minimizes bias from outliers and ensures reliable correlation estimates. Artwork of human shape and tissues: Created in BioRender. Bolis, M. (2025) https://BioRender.com/d53m930

SUPPLEMENTARY FIGURE 2

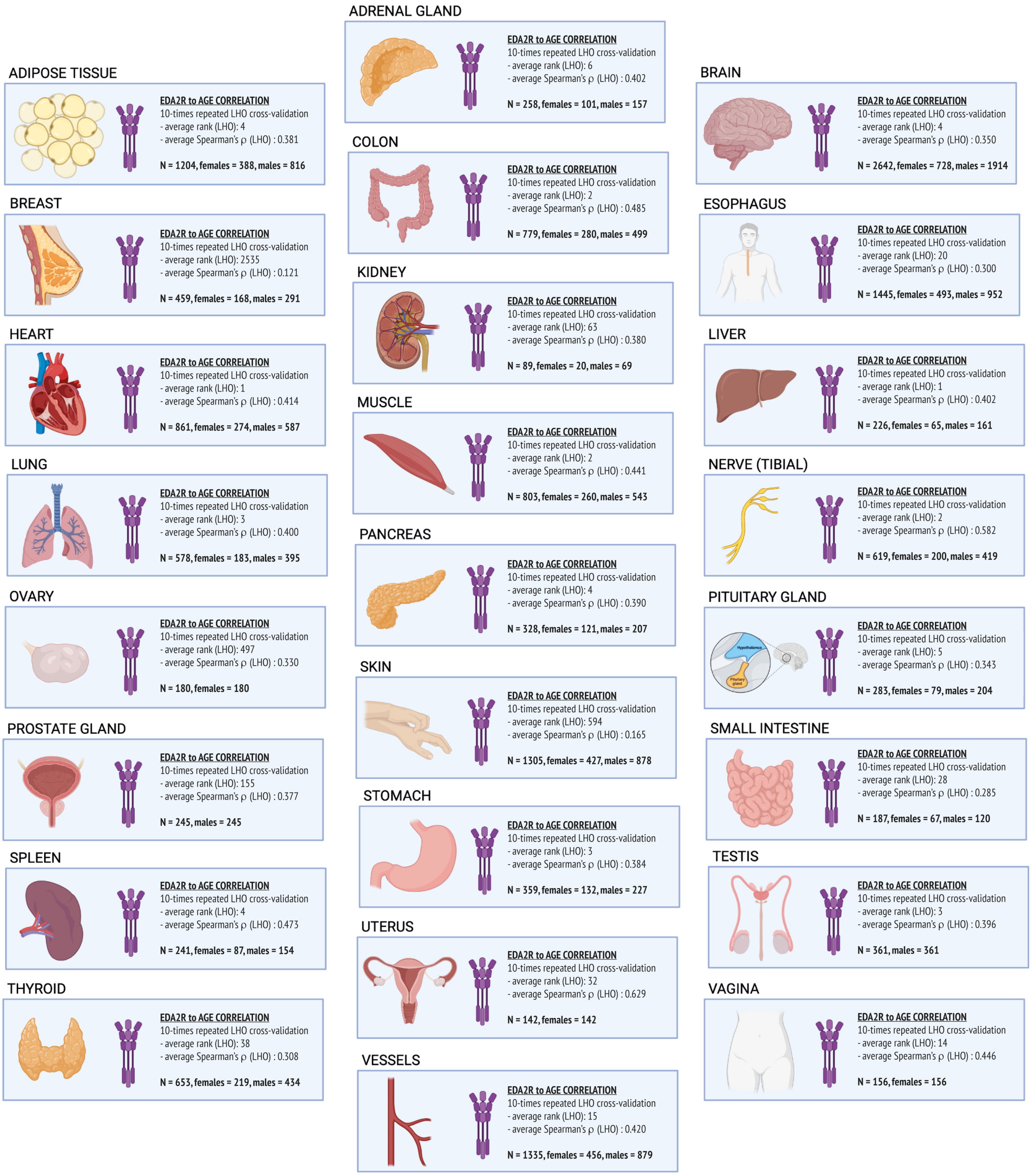

Association of EDA2R with age in human tissues

Correlation between *EDA2R* gene expression and age across human tissues from the GTEX dataset as determined from 10-fold repeated leave-half-out (LHO) cross-validation procedure. Average ranking across LHO subsets, average Spearman's correlation coefficient (ρ), total and gender-specific sample sizes are provided for each tissue. Within each tissue, gene expression values were batch-adjusted for sex and for additional anatomical sub localization. The source data are contained in the Source Data file. Created in BioRender. Bolis, M. (2025) https://BioRender.com/p21q902

SUPPLEMENTARY FIGURE 3

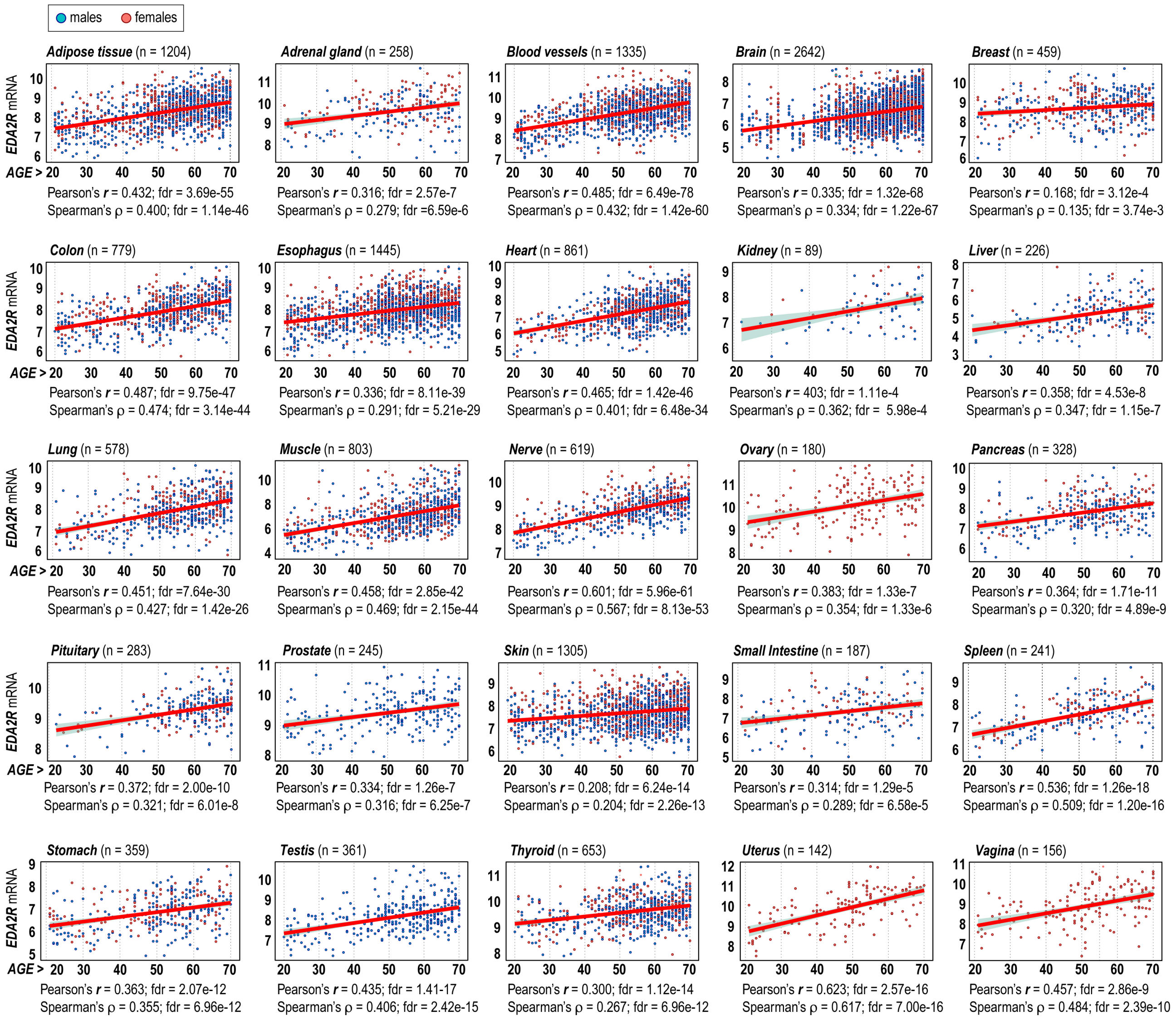

Correlation between EDA2R and age in human tissues

Scatterplots showing tissue-specific correlations between *EDA2R* expression (vst-normalized) and age in human samples from the GTEx dataset. Pearson's and Spearman's correlation coefficients are provided, along with their associated FDR-adjusted p-values. Within each tissue, gene expression values were batch-adjusted for additional anatomical sub localization but not for sex, to preserve sex-associated differences. Outliers, defined as samples with *EDA2R* expression values greater or less than 3 standard deviations from the mean, were excluded. Source data are contained in the Source Data file.

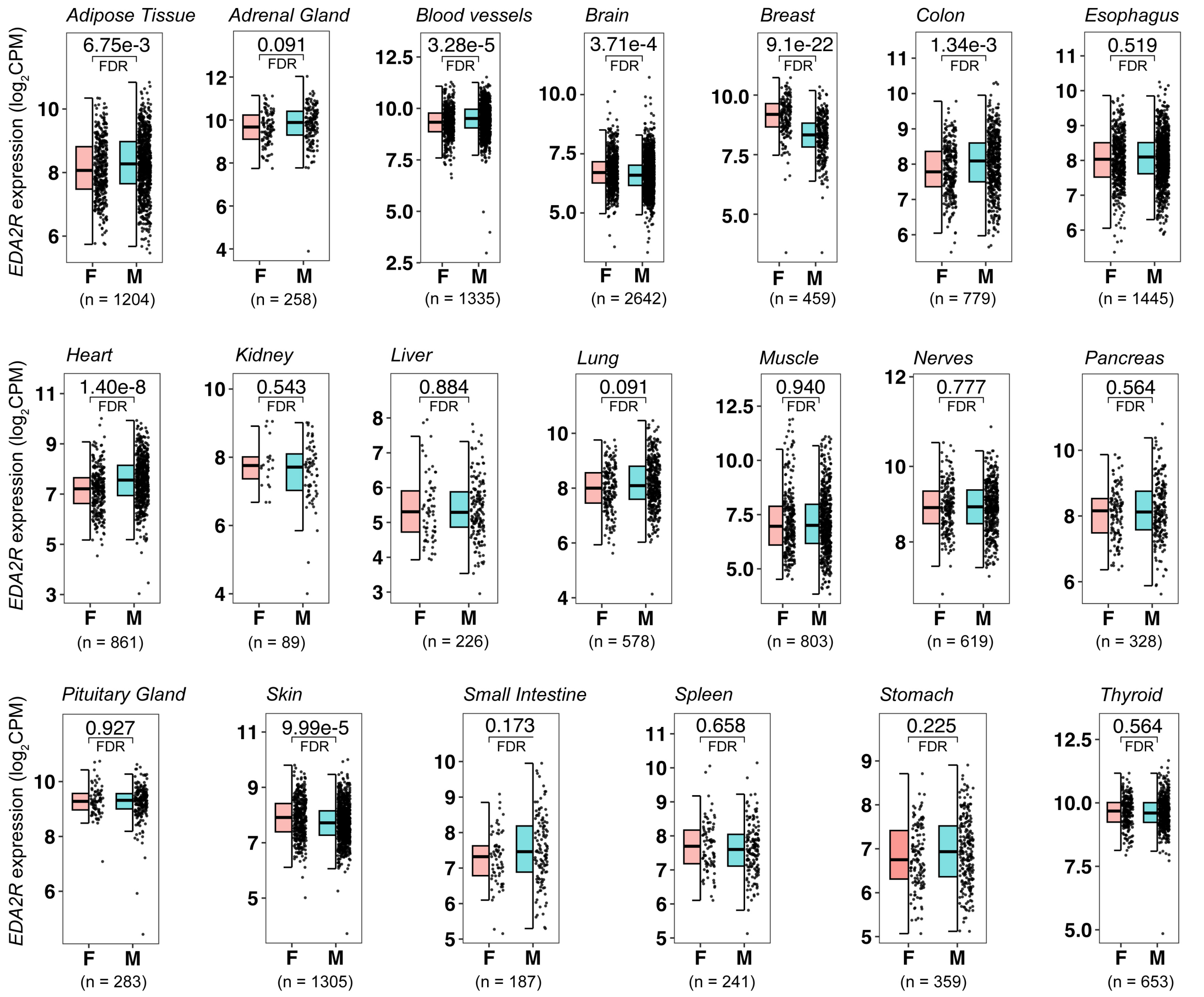

Sex-specific differences in *EDA2R* mRNA levels

Boxplots showing sex-specific differences in *EDA2R* expression between females (F; red) and males (M; blue) across tissues (GTEX dataset). Within each tissue, gene expression values were batch-adjusted for additional anatomical sub localization but not for sex, to preserve sex-associated differences. Statistical significance was assessed using two-tailed Wilcoxon rank sum test and adjusted for multiple testing using FDR. Source data and boxplots boundaries are provided in Source Data File.

SUPPLEMENTARY FIGURE 5

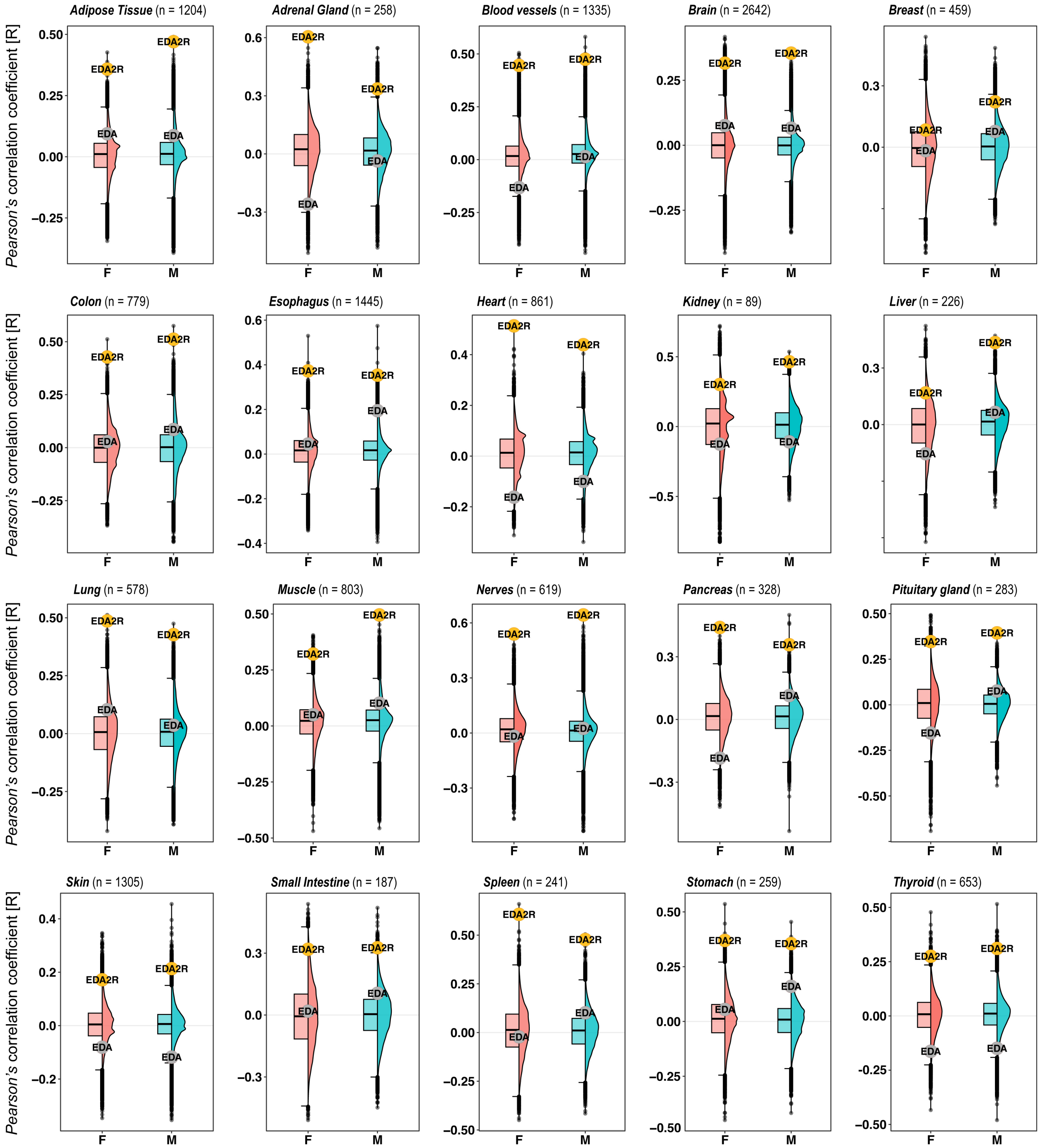

Correlation between age and *EDA2R*/*EDA* expression in males and females

Boxplots showing correlations between age and *EDA2R*, *EDA* expression separately in females (F, red) and males (M, blue) across tissues (GTEX dataset). Within each tissue, gene expression values were batch-adjusted for additional anatomical sub localization but not for sex, to preserve sex-associated differences. Correlations are quantified using Pearson's coefficients. Source data and boxplots boundaries are provided in Source Data File.

SUPPLEMENTARY FIGURE 6

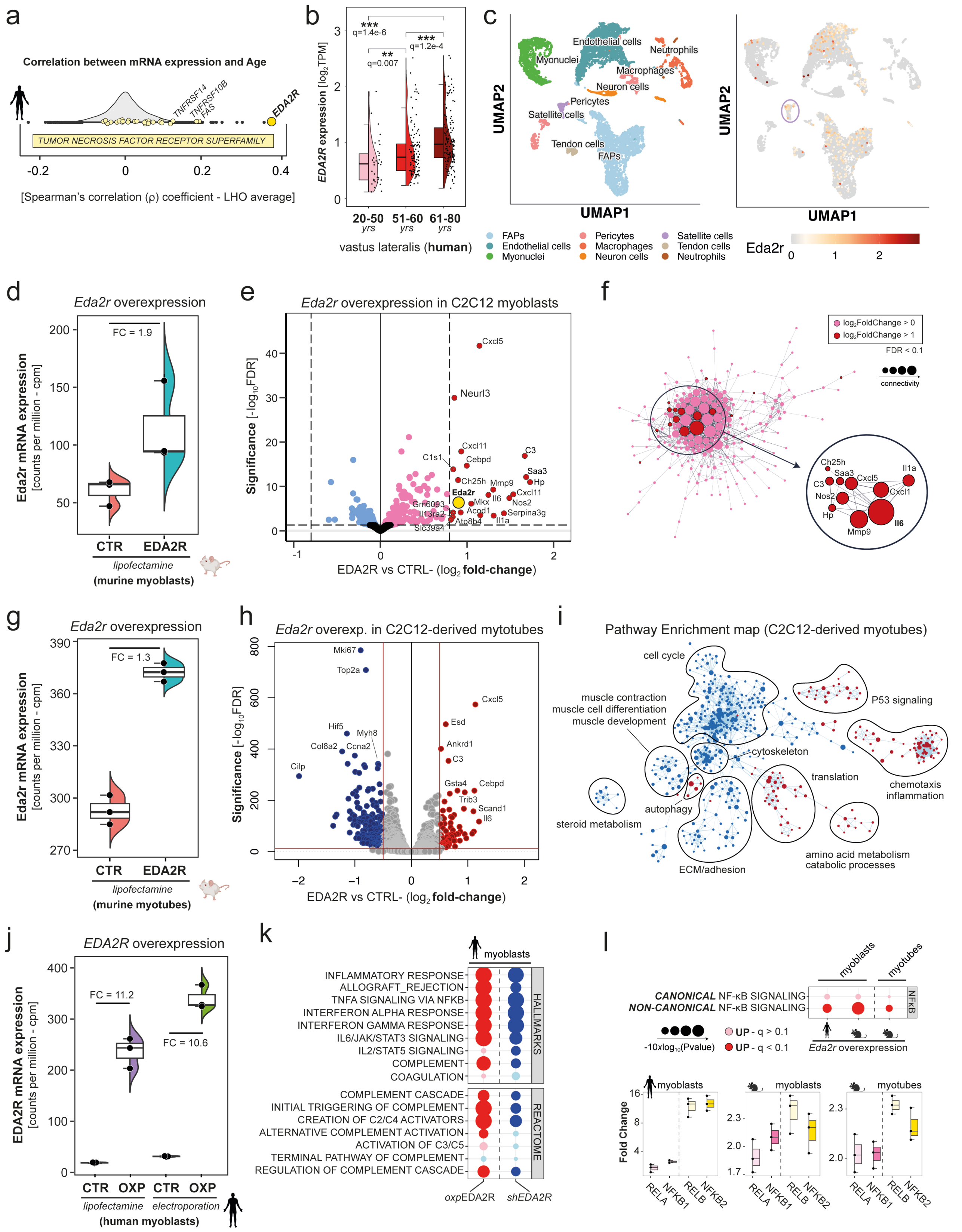

Functional Experiments in Myoblast and Myotube Models

a) Violin plot depicting the distribution of median Pearson's correlation coefficients between age and mRNA expression across tissues (GTEX dataset). Indicated are *EDA2R* and all other annotated Tumor Necrosis Factor receptors in humans, including death-domain containing receptors (*TNFRSF1A, FAS, TNFRSF25, TNFRSF10A, TNFRSF10B, TNFRSF21*), TRAF-interacting motif containing receptors (*TNFRSF1B, CD40, TNFRSF8, CD27, LTBR, TNFRSF4, TNFRSF9, TNFRSF13C, TNFRSF17, TNFRSF13B, TNFRSF11A, TNFRSF16, TNFRSF14, TNFRSF18, TNFRSF19, EDAR, EDA2R, RELT, TNFRSF12A*), and tethering receptors (*TNFRSF10C, TNFRSF10D, TNFRSF6B, TNFRSF11B*). Human shape: Created in BioRender. Bolis, M. (2025) https://BioRender.com/w99k679. **b)** Boxplots illustrating mRNA expression (TPM) of *EDA2R* in human samples derived from vastus lateralis muscle (n=291, phs001048). Patients were categorized into 3 groups based on their age. Darker shades of red represent older age groups. P-values were determined using Wilcoxon rank sum test (two-sided) and adjusted for multiple comparison using FDR. (age group 20-50 n=34; 51-60 n = 110; 61-80 n = 147). Individuals aged 20 to 50 were aggregated to account for the underrepresentation of the 20-40 age group. In each group, samples with expression values greater than 3 standard deviations from the mean were discarded. Exact q-values are provided (*** q ≤ 001=; ** q ≤ 0.01; * q ≤ 0.05). **c)** UMAP showing single cells from murine skeletal muscles, encompassing 11,734 cells as quantified by Zhang et al. (2022). **Left:** Major cell types annotated. **Right**: Expression levels of *EDA2R* across cell-types. **d)** Boxplots comparing gene-expression (counts per million) in control (red) vs *Eda2r* (lightblue) overexpressing C2C12 murine myoblasts, each group consisting of n=3 biological replicates. Fold-induction = 1.9; FDR = 8.59e-07 (computed using two-tailed Wald statistics as implemented in DESeq2, p-values adjusted for multiple testing using FDR). Mouse shape: Created in BioRender. Bolis, M. (2025) https://BioRender.com/m04r083 **e)** Volcano-plot depicting differential expression in C2C12 myoblasts overexpressing *Eda2r* versus GFP overexpressing controls. Indicated in light blue are significantly down-regulated (log_2 fold-change<0) genes with an FDR < 0.05. Upregulated genes (FDR<0.05) are indicated in pink (log_2 fold-change>0) or red (log_2 fold-change>0.8). P-Values were computed using two-tailed Wald statistics as implemented in DESeq2, and adjusted for multiple testing using FDR**. f)** Protein-protein interaction network (String-DB) constructed using significantly up-regulated genes from d). **g)** Boxplots comparing gene-expression (counts per million) in C2C12-derived myotubes overexpressing *Eda2r* (lightblue) or GFP (red), each group consisting of 3 biological replicates. Fold-induction = 1.3; FDR = 2.50e-15 (computed using two-tailed Wald statistics as implemented in DESeq2, and adjusted for multiple testing using FDR). Mouse shape: Created in BioRender. Bolis, M. (2025) https://BioRender.com/g10m187 **h)** Volcano-plot depicting differential expression of C2C12-derived myotubes overexpressing *Eda2r* versus GFP controls. Blue and red colors indicate down or upregulated genes with a log_2 fold-change lower than -0.5 or greater than 0.5 and an FDR<0.05. **i)** Enrichment map depicting significant induction or repression of gene sets from the comparison of transcriptional profiles of *EDA2R*-overexpressing vs. wild-type murine C2C12-differentiated into myotubes. Each dot represents an individual gene set, with the size of dots proportional to the size of each gene set. Edges between nodes represent shared genes between adjacent pathways. The analysis includes gene sets from the Hallmark, Reactome, Wikipathways, and GeneOnotlogy (BP-biological processes) collections. **j)** Boxplots comparing gene-expression (counts per million) in control vs *EDA2R* overexpressing human myoblasts transfected either by lipofectamine (violet; fold-induction = 11.2; FDR < 2.47e-324, n = 3 biological replicates per group) or electroporation (green; fold-induction = 10.6; FDR < 2.47e-324, n = 3 biological replicates per group). Adjusted p-values were computed using Wald statistics as implemented in DESeq2. Human shape: Created in BioRender. Bolis, M. (2025) https://BioRender.com/m53w722 **k)** Enriched inflammation-related gene sets from the Hallmarks and Reactome collections, as determined in human myoblasts following overexpression (column 1) or knockdown (columns 2) of *EDA2R*. Red and blue dots indicate up and downregulated gene-sets, respectively. P-Values were adjusted for multiple testing using FDR. Human shape: Created in BioRender. Bolis, M. (2025) https://BioRender.com/w75o822 **l)** Enrichment analysis (top) and fold-change increase (bottom, n = 3 biological replicates per group) of key genes belonging to the canonical (*NFKB1+RELA*) and non-canonical (*NFKB2+RELB*) NF-κB signaling pathways, following overexpression of *EDA2R*. Enrichments results and gene-expression fold-changes are presented for human myoblasts, C2C12 murine myoblasts, and C2C12-differentiated murine myotubes. P-Values were adjusted for multiple testing using FDR. (color codes: lightpink = *RELA*; *NFKB1* = pink; *RELB* = lightyellow; *NFKB2* = yellow). Human and mouse shapes: Created in BioRender. Bolis, M. (2025) https://BioRender.com/t75h103

SUPPLEMENTARY FIGURE 7

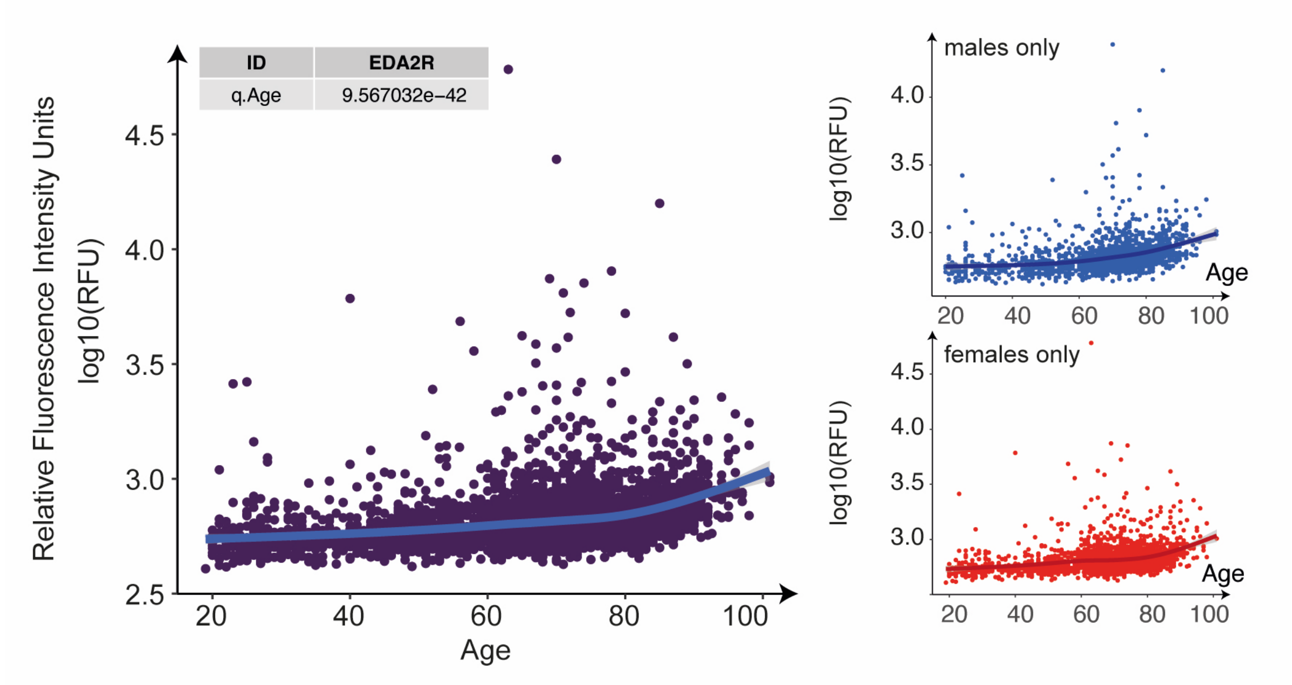

Correlation between age and EDA2R protein abundance in males and females

Scatterplots showing correlation between EDA2R abundance and age. Protein abundance was determined in plasma samples from 5,676 adults as described in (10.1038/s41586-023-06802-1) (https://twc-stanford.shinyapps.io/aging_plasma_proteome_v2). (color codes: navy = males and females combined; blue = males only; red = females only).

SUPPLEMENTARY FIGURE 8

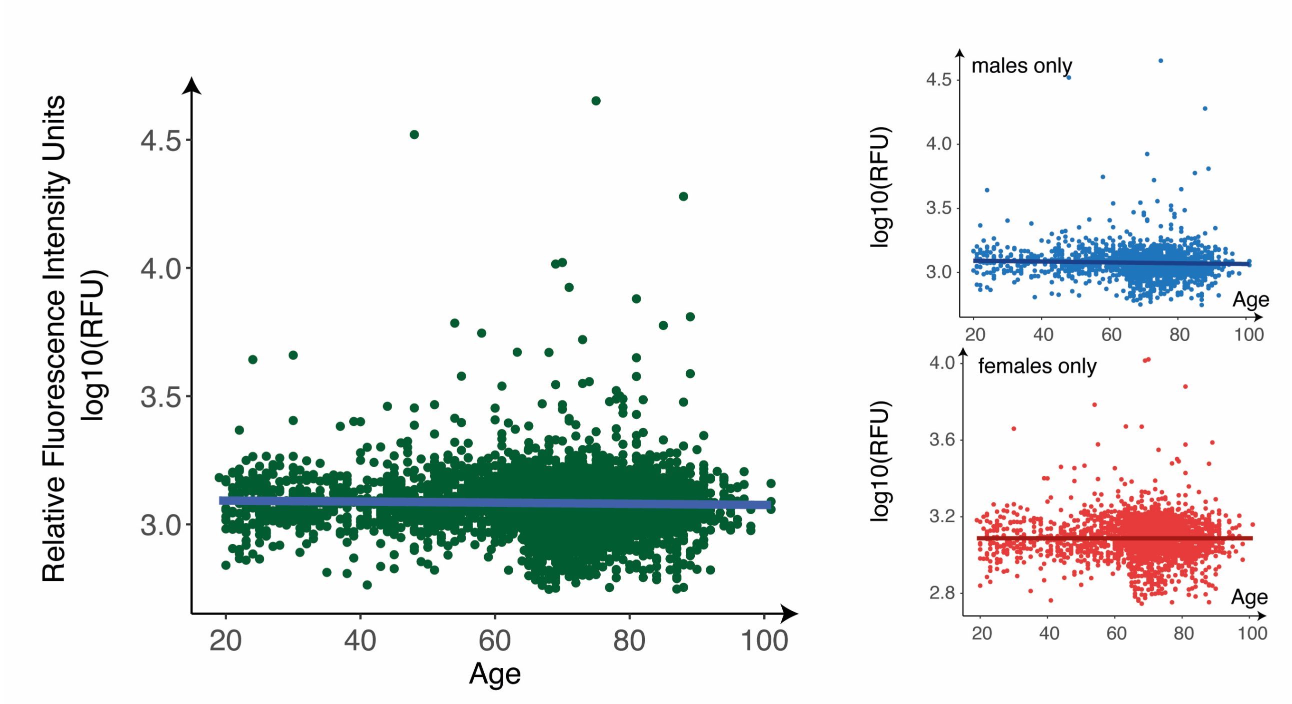

Correlation between age and EDA protein abundance in males and females

Scatterplots showing correlation between EDA abundance and age. Protein abundance was determined in plasma samples from 5,676 adults as described in (10.1038/s41586-023-06802-1) (https://twc-stanford.shinyapps.io/aging_plasma_proteome_v2). (color codes: green = males and females combined; blue = males only; red = females only).
