# Supplementary material for: Increased ectodysplasin-A2-receptor EDA2R is a ubiquitous hallmark of aging and mediates parainflammatory responses
Source: Nat Commun. 2025 Feb 23;16:1898. doi: 10.1038/s41467-025-56918-3 (PMC11847917; doi:10.1038/s41467-025-56918-3)
Supplement: Supplementary file 2 — Description of Additional Supplementary Files [file 41467_2025_56918_MOESM2_ESM.pdf]

# <u>Description of Additional Supplementary Files</u>

**Supplementary Data 1:**

Reported are average Spearman's correlation coefficients between geneexpression and chronological age of donor (human) as determined by 10-fold Leave Half Out random resampling.

**Supplementary Data 2:**

P-values determined from n=1000 permutations (correlations between geneexpression and age)

**Supplementary Data 3:**

Pearson's correlation coefficients between gene-expression and chronological age of mice across 14 different tissues.

**Supplementary Data 4:**

Pearson's correlation coefficients between gene-expression and chronological age of

rats across 11 different tissues.

**Supplementary Data 5:**

Differential expression results of HGPS mouse models compared to aged-matched

wild-type counterpart (aortic artery -GSE165409).

**Supplementary Data 6:**

Quantification of EDA2R mRNA expression in human vastus lateralis muscle samples from dbGap (phs001048).

**Supplementary Data 7:**

Differential expression results from microarray experiment (GSE52550), comparing the gastrocnemius muscle of aged mice to that of young mice.

**Supplementary Data 8:**

Differential expression results (GSE53960) of Eda2r mRNA expression changes in

gastrocnemius muscle of aged rats compared to baseline (6 months)

**Supplementary Data 9:**

Murine C2C12 myoblasts transfected with Eda2r vs matched controls (GFP)

**Supplementary Data 10:**

Murine C2C12-derived myotubes overexpressing Eda2r vs matched controls

(GFP)

**Supplementary Data 11:**

Human primary myoblasts transfected with EDA2R vs matched controls (GFP)

**Supplementary Data 12:**

Human primary myoblasts supplemented with EDA-A2 vs vehicle

**Supplementary Data 13:**

Isoform-level expression of EDA-A2 quantified in GSE107011 (Monaco et al), a reference for annotating blood cells across 29 cell types.
