# Supplementary material for: Increased ectodysplasin-A2-receptor EDA2R is a ubiquitous hallmark of aging and mediates parainflammatory responses
Source: Nat Commun. 2025 Feb 23;16:1898. doi: 10.1038/s41467-025-56918-3 (PMC11847917; doi:10.1038/s41467-025-56918-3)
Supplement: Supplementary file 16 — Reporting Summary [file 41467_2025_56918_MOESM16_ESM.pdf]

nature portfolio

Corresponding author(s): MARCO BOLIS

Last updated by author(s): Jan 13, 2025

Reporting Summary

Nature Portfolio wishes to improve the reproducibility of the work that we publish. This form provides structure for consistency and transparency in reporting. For further information on Nature Portfolio policies, see our Editorial Policies and the Editorial Policy Checklist.

Statistics

For all statistical analyses, confirm that the following items are present in the figure legend, table legend, main text, or Methods section.

n/a	Confirmed	
☐	☒	The exact sample size (*n*) for each experimental group/condition, given as a discrete number and unit of measurement
☐	☒	A statement on whether measurements were taken from distinct samples or whether the same sample was measured repeatedly
☐	☒	The statistical test(s) used AND whether they are one- or two-sided<br>*Only common tests should be described solely by name; describe more complex techniques in the Methods section.*
☐	☒	A description of all covariates tested
☐	☒	A description of any assumptions or corrections, such as tests of normality and adjustment for multiple comparisons
☐	☒	A full description of the statistical parameters including central tendency (e.g. means) or other basic estimates (e.g. regression coefficient) AND variation (e.g. standard deviation) or associated estimates of uncertainty (e.g. confidence intervals)
☐	☒	For null hypothesis testing, the test statistic (e.g. *F*, *t*, *r*) with confidence intervals, effect sizes, degrees of freedom and *P* value noted<br>*Give P values as exact values whenever suitable.*
☒	☐	For Bayesian analysis, information on the choice of priors and Markov chain Monte Carlo settings
☒	☐	For hierarchical and complex designs, identification of the appropriate level for tests and full reporting of outcomes
☐	☒	Estimates of effect sizes (e.g. Cohen's *d*, Pearson's *r*), indicating how they were calculated

Our web collection on statistics for biologists contains articles on many of the points above.

Software and code

Policy information about availability of computer code

Data collection	no specific software was used to collect data
Data analysis	Analyses were performed in the R statistical environment (v4.2.3), and sequencing counts were normalized and further processed using DESeq2 (v1.38.3). Batch effects were corrected using the limma package (v3.54.2). Gene expression from the phs001048.v2.p1 dataset was calculated using htseq-count (v0.5.46) with the basic GENCODE v19 annotation and presented in transcripts per kilobase million (TPM). Analysis of high-throughput experiments involving human myoblasts was performed by mapping sequencing reads to the human genome (GRCh38) using the STAR aligner (v2.7.9a) and Gencode (v38) annotations. Analysis of high-throughput experiments involving murine myoblasts and myotubes was performed by mapping sequencing reads to the mouse genome (GRCm39) using the STAR aligner (v2.7.9a) and Gencode (M27) annotations. Gene-set enrichment analyses used for dot plot representation were performed with Camera (limma v3.54.2). Pathway enrichment maps were generated on top of Gene Set Enrichment Analysis results (GSEA v4.3.2) and represented using Cytoscape (v3.9.1). Affymetrix Mouse Genome 430 2.0 Arrays were reprocessed from raw files using the affy (v1.76.0) package and normalized with the Robust Multi-Array expression measure using sequence information provided by the gcrma (v2.70.0) package. Probeset-level analysis of microarray experiments of murine models of HPGS syndrome involving keratinocytes (GSE67288) and gonadal adipocytes (GSE51203) was processed in the R statistical environment using the limma (v3.54.2) package. Additional code to facilitate the replication of results obtained for human subjects, animal, and experimental models is available on our GitHub repository: https://github.com/BolisLab/eda2r.

For manuscripts utilizing custom algorithms or software that are central to the research but not yet described in published literature, software must be made available to editors and reviewers. We strongly encourage code deposition in a community repository (e.g. GitHub). See the Nature Portfolio guidelines for submitting code & software for further information.

Data

Policy information about availability of data

All manuscripts must include a data availability statement. This statement should provide the following information, where applicable:

- Accession codes, unique identifiers, or web links for publicly available datasets
- A description of any restrictions on data availability
- For clinical datasets or third party data, please ensure that the statement adheres to our policy

Raw sequencing data generated throughout this study are available from EBI-Biostudies (ArrayExpress) under the accession numbers E-MTAB-13101, E-MTAB-14234, and E-MTAB-14239. A source data file containing the numeric data used to generate the figure panels is deposited along with the manuscript.
Specifically:
- E-MTAB-13101: Eda2r overexpression in C2C12 murine myoblasts
- E-MTAB-14234: Eda2r overexpression in murine C2C12 cells differentiated into myotubes
- E-MTAB-14239: Experiments performed in human myoblasts involving EDA2R transfection by either lipofectamine or electroporation, treatment with the EDA-A2 ligand, and silencing of EDA2R through the action of short hairpins.

RNA-Seq raw count data were retrieved from GTEx v8, which includes 17,382 samples from 980 healthy individuals. Data from the muscle biopsy study (FUSION) were used with permission (Database of Genotypes and Phenotypes dbGaP Study Accession: phs001048.v2.p1). Processed single-cell sequencing data of human skeletal muscles were downloaded from the Human Muscle Ageing Cell Atlas (https://db.cngb.org/cdcp/hlma). Processed single-cell sequencing data of mouse skeletal muscles were downloaded from the Dryad repository (10.5061/dryad.t4b8gtj34) and from Zhang et al. (https://mayoxz.shinyapps.io/Muscle). Gene expression data derived from multiple tissues of mice (adipose tissue, brain, liver, lung, limb, small intestine, spleen, pancreas, heart, kidney, skin) and rats (adrenal gland, brain, heart, kidney, lung, skeletal muscle, spleen, thymus, testes, pancreas, uterus) were retrieved from GEO (GSE132040 and GSE53960, respectively). Microarray-derived gene expression data (Affymetrix Mouse Genome 430 2.0 Array) for the gastrocnemius muscle of young (10 weeks) and old (24 months) mice were retrieved from GEO (GSE52550). RNA-Seq data for the gastrocnemius muscle of rats were downloaded from the SRA project PRJNA516151. Microarray experiments of murine models of HPGS syndrome involving keratinocytes (GSE67288) and gonadal adipocytes (GSE51203) were downloaded from GEO. EDA2R expression in blood from the NESDA and NTR studies was retrieved with permission from (phs000486).

Research involving human participants, their data, or biological material

Policy information about studies with human participants or human data. See also policy information about sex, gender (identity/presentation), and sexual orientation and race, ethnicity and racism.

Reporting on sex and gender	Skeletal muscle myoblasts were isolated from a human male donor. Written informed consent was obtained from the donor, and the study protocol was approved by the Ethical Review Committee at the Ludwig-Maximilians-University, Munich, Germany (IRB-No. 45-14).<br>We did not perform any clinical trial or additional study involving the recruitment of patients. We analyzed existing databases containing human gene expression data such as GTEx or FUSION (phs001048.v2.p1). Experiments involving human cells in vitro were performed on human myoblasts obtained from a male donor, as described in the methods section.<br>Sex information was considered for both the GTEx and FUSION (phs001048.v2.p1) datasets. Information regarding sex is provided in supplementary files, and analyses stratified by sex were presented in the manuscript and supplementary figures.<br>When investigating the transcriptomic output, we determined that sex was the major source of biological variation within the transcriptional output of human samples (GTEx), as demonstrated by PCA analyses.
Reporting on race, ethnicity, or other socially relevant groupings	The GTEX dataset includes a diverse mix of ethnicities and genders, reflecting U.S. demographics. This information is available at the study web page (https://gtexportal.org/home/).<br>Race, ethnicity, or other socially relevant grouping did not have a major impact in our analysis. Indeed, when investigating the transcriptomic output, we determined that sex was the major source of biological variation within the transcriptional output of human samples (GTEx), as demonstrated by PCA analyses.
Population characteristics	The GTEx dataset comprises data from approximately 948 donors, aged 20-70. Donors were generally healthy individuals who died from sudden, non-disease-related causes, minimizing disease-related confounding factors. The dataset includes over 17,000 tissue samples from more than 50 tissue types, capturing broad genetic and environmental diversity to study the relationship between genetic variation and gene expression across different human tissues.
Recruitment	We did not perform any clinical trial or study involving the recruitment of patients.
Ethics oversight	We did not perform any clinical trial or study involving the recruitment of patients.

Note that full information on the approval of the study protocol must also be provided in the manuscript.

Field-specific reporting

Please select the one below that is the best fit for your research. If you are not sure, read the appropriate sections before making your selection.

☒ Life sciences ☐ Behavioural & social sciences ☐ Ecological, evolutionary & environmental sciences

For a reference copy of the document with all sections, see nature.com/documents/nr-reporting-summary-flat.pdf

Life sciences study design

All studies must disclose on these points even when the disclosure is negative.

Sample size	In our cell line experiments, we conducted three biological replicates per condition. This decision was based on the observed variance between conditions, which was significantly greater than the variance within the same experimental group. To ensure the robustness of our findings, we performed statistical analyses to confirm that this sample size was adequate to detect meaningful differences between conditions. Specifically, we used a power analysis to determine that 3 biological replicates was sufficient to achieve a statistical power of 0.8, with an alpha level of 0.05.
Data exclusions	no data were excluded
Replication	In our transcriptomics data produced for myoblasts and myotubes (human or mice), three biological replicates per condition were sufficient, as gene expression analysis showed that the variability within experimental groups far exceeded the variability between individual samples, as clearly seen in principal component analysis. This is particularly relevant since we are working with cell lines, which exhibit lower variability compared to in vivo models. Our experiments were replicated across different cell types (myoblasts and myotubes) and species (mice and humans), consistently yielding similar results. Moreover, our differential expression analysis revealed highly statistically significant results using false discovery rate (FDR) adjustments, underscoring the robustness and reliability of our data.
Randomization	We performed differential expression experiments between known experimental groups, such as EDA2R overexpression vs. GFP or EDA-A2 treatment vs. Vehicle; therefore, there was no need for randomization.
Blinding	We performed differential expression experiments between known experimental groups, such as EDA2R overexpression vs. GFP or EDA-A2 treatment vs. Vehicle; therefore, blinding was not necessary.

Reporting for specific materials, systems and methods

We require information from authors about some types of materials, experimental systems and methods used in many studies. Here, indicate whether each material, system or method listed is relevant to your study. If you are not sure if a list item applies to your research, read the appropriate section before selecting a response.

Materials & experimental systems

n/a	Involved in the study	
☒	☐	Antibodies
☐	☒	Eukaryotic cell lines
☒	☐	Palaeontology and archaeology
☒	☐	Animals and other organisms
☒	☐	Clinical data
☒	☐	Dual use research of concern
☒	☐	Plants

Methods

n/a	Involved in the study	
☒	☐	ChIP-seq
☒	☐	Flow cytometry
☒	☐	MRI-based neuroimaging

Eukaryotic cell lines

Policy information about cell lines and Sex and Gender in Research

Cell line source(s)	C2C12 myoblast cell line from the C3H mouse strain (male), was obtained from ATCC (number #CRL-1772). Skeletal muscle myoblasts were isolated from a human male donor with consent and provided by Prof. Schoser (University of Munich), who is among the coauthors of this manuscript.
Authentication	Cell lines were directly purchased from ATCC, and their transcriptional output clusters together with gene expression obtained for C2C12 cells from external datasets. Additionally, C2C12 cells have peculiar characteristics of myoblasts, such as their ability to form myotubes.
Mycoplasma contamination	Cell lines were routinely tested and found to be negative for mycoplasma.
Commonly misidentified lines (See ICLAC register)	none

Plants

Seed stocks	we did not make use of plants
Novel plant genotypes	we did not make use of plants
Authentication	we did not make use of plants
